# Genome-Wide Analysis of Copy Number Variation Identifies Candidate Gene Loci Associated with the Progression of Non-Alcoholic Fatty Liver Disease

**DOI:** 10.1371/journal.pone.0095604

**Published:** 2014-04-17

**Authors:** Shamsul Mohd Zain, Rosmawati Mohamed, David N. Cooper, Rozaimi Razali, Sanjay Rampal, Sanjiv Mahadeva, Wah-Kheong Chan, Arif Anwar, Nurul Shielawati Mohamed Rosli, Anis Shafina Mahfudz, Phaik-Leng Cheah, Roma Choudhury Basu, Zahurin Mohamed

**Affiliations:** 1 The Pharmacogenomics Laboratory, Faculty of Medicine, University of Malaya, Kuala Lumpur, Malaysia; 2 Department of Pharmacology, Faculty of Medicine, University of Malaya, Kuala Lumpur, Malaysia; 3 Department of Medicine, Faculty of Medicine, University of Malaya, Kuala Lumpur, Malaysia; 4 Institute of Medical Genetics, School of Medicine, Cardiff University, Cardiff, United Kingdom; 5 Sengenics Sdn Bhd, High Impact Reseach Building, University of Malaya, Kuala Lumpur, Malaysia; 6 Julius Centre University of Malaya, Department of Social and Preventive Medicine, Faculty of Medicine, University of Malaya, Kuala Lumpur, Malaysia; 7 Medical Imaging Unit, Faculty of Medicine, University of Technology MARA, Sungai Buloh Campus, Selangor, Malaysia; 8 Department of Pathology, Faculty of Medicine, University of Malaya, Kuala Lumpur, Malaysia; 9 Clinical Investigation Centre, University Malaya Medical Centre, Kuala Lumpur, Malaysia; Northeast Ohio Medical University, United States of America

## Abstract

Between 10 and 25% of individuals with non-alcoholic fatty liver disease (NAFLD) develop hepatic fibrosis leading to cirrhosis and hepatocellular carcinoma (HCC). To investigate the molecular basis of disease progression, we performed a genome-wide analysis of copy number variation (CNV) in a total of 49 patients with NAFLD [10 simple steatosis and 39 non-alcoholic steatohepatitis (NASH)] and 49 matched controls using high-density comparative genomic hybridization (CGH) microarrays. A total of 11 CNVs were found to be unique to individuals with simple steatosis, whilst 22 were common between simple steatosis and NASH, and 224 were unique to NASH. We postulated that these CNVs could be involved in the pathogenesis of NAFLD progression. After stringent filtering, we identified four rare and/or novel CNVs that may influence the pathogenesis of NASH. Two of these CNVs, located at 13q12.11 and 12q13.2 respectively, harbour the exportin 4 (*XPO4*) and phosphodiesterase 1B (*PDE1B*) genes which are already known to be involved in the etiology of liver cirrhosis and HCC. Cross-comparison of the genes located at these four CNV loci with genes already known to be associated with NAFLD yielded a set of genes associated with shared biological processes including cell death, the key process involved in ‘second hit’ hepatic injury. To our knowledge, this pilot study is the first to provide CNV information of potential relevance to the NAFLD spectrum. These data could prove invaluable in predicting patients at risk of developing NAFLD and more importantly, those who will subsequently progress to NASH.

## Introduction

Non-alcoholic fatty liver disease (NAFLD) has emerged as a silent epidemic, with its worldwide prevalence continuing to increase with the growing incidence of obesity [1]. NAFLD comprises a spectrum of diseases ranging from simple steatosis, which is essentially benign fatty infiltration of the liver, to its inflammatory counterpart non-alcoholic steatohepatitis (NASH) [2]. The pathogenesis of NAFLD is based on the “two hit hypothesis” [3]. The “first hit” is the development of steatosis and involves the accumulation of triglycerides in the liver due to insulin resistance. Insulin resistance prepares the hepatocytes for the second insult. The “second hit” is often due to adipocytokines and oxidative stress, which further damage the liver thereby promoting progression to steatohepatitis and fibrosis. A significant proportion of individuals with NAFLD develop hepatic fibrosis, a key feature of the condition which is associated with progression of the disease to cirrhosis and its related complications, including hepatic failure and hepatocellular carcinoma [4]. The fibrotic progression of NAFLD is identified histologically by the presence of NASH. A high prevalence of NASH is found among those with insulin resistance-related comorbidities such as obesity and type 2 diabetes [5]. The mortality rate among NASH patients has been found to be much higher than for patients with simple fatty liver (simple steatosis) [6].

In addition to environmental factors such as high calorific food intake and a sedentary lifestyle, there is mounting evidence of a genetic component to the complex etiology of NAFLD [7]. This is reflected by marked differences in the prevalence of NAFLD across diverse populations [8]–[9]. The high heritability of NAFLD was evident in a familial aggregation study, with estimates of 59% in siblings and 78% in parents with NAFLD [10]. Until recently, genome-wide association studies (GWAS) and the candidate gene approach have both utilised single nucleotide polymorphisms (SNPs) to explain the genetic component of NAFLD [7], [11]–[12].

The wide distribution of copy number variants (CNVs) in the human genome has underscored the importance of CNVs in relation to genetic diversity, phenotypic variability and disease susceptibility [13]–[14]. It has been estimated that approximately 12% of the human genome is copy number variable [15] with over 1000 genes having been mapped within or close to regions that are affected by structural variation [16]. A global increase in CNV burden has also been observed in polygenic traits such as schizophrenia [17], autism [18] and attention deficit hyperactivity disorder [19]. Given these findings, the sheer scale of CNVs means that they are likely to make a significant contribution to the ‘missing heritability’ of some of these conditions [20]. However, despite some success in identifying CNVs responsible for metabolic phenotypes including obesity and diabetes mellitus [21]–[22], there are as yet no data available to suggest whether or not CNVs might be involved in the etiology of the NAFLD spectrum.

Here, we describe a pilot study designed to detect rare or novel CNVs associated with NAFLD and/or NASH. Predicting NASH non-invasively is very important since this condition is potentially progressive and liver biopsy is currently the gold standard for the diagnosis of NASH. We interrogated the CNVs associated with NASH and ascertained the biological processes associated with those genes covered by the CNVs in order to assess their possible role in the progression of the disease. To this end, we used a high-resolution Agilent aCGH platform to perform genome-wide copy number analysis in patients with both simple steatosis and NASH, which are representative of the clinical spectrum of NAFLD.

## Materials and Methods

### Ethics Statement

The study protocol was approved by the Medical Ethics Committee of UMMC and all subjects provided their written informed consent to participate.

### Subjects

Genome-wide copy number profiling was performed using array comparative genomic hybridization (aCGH) on a total of 49 NAFLD patients (39 with NASH and 10 with simple steatosis) and 49 fatty liver-free controls that were matched both for age and gender. All subjects were, as far as could ascertain, genetically unrelated to each other. All NAFLD patients were consecutively recruited from the University of Malaya Medical Centre (UMMC). NAFLD was confirmed through liver histology and evaluated according to the NASH Clinical Research Network criteria [23]–[24]. All liver biopsy specimens were on average 1.5 cm long and contained at least six portal tracts. Subjects were excluded if they met any of the following criteria: (i) alcohol consumption >10g/day [25]; (ii) hepatitis B or C infection; (iii) autoimmune hepatitis; (iv) exposure to drugs known to cause steatosis or (v) Wilson’s disease. The controls were genetically unrelated healthy subjects with a body mass index (BMI) <25 kg/m^2^, a fasting plasma glucose of <110 mg/dL, a normal lipid profile and normal liver enzymes. NAFLD was actively excluded in the controls by ultrasonography according to the absence of the following criteria: (i) slight diffuse increase in bright homogeneous echoes in the liver parenchyma with normal visualization of the diaphragm and portal and hepatic vein borders, and normal hepatorenal echogenicity contrast; (ii) diffuse increase in bright echoes in the liver parenchyma with slightly impaired visualization of the peripheral portal and hepatic vein borders; (iii) marked increase in bright echoes at a shallow depth with deep attenuation, impaired visualization of the diaphragm and marked vascular blurring [26]. Subsequent magnetic resonance imaging (MRI) to further confirm the fatty liver free status was performed.

### Array CGH

Array-CGH was performed according to the protocol established by the manufacturer (Oxford Gene Technology, Begbroke, UK). It was carried out using the SurePrint G3 Human CGH 2×400 K array (Agilent Technologies, Santa Clare, CA, USA) for genome-wide identification of putative disease-associated CNVs. Each oligonucleotide-based microarray slide contained 410,739 probes that enabled the profiling of molecular genomic imbalances with a mean resolution of 5.3 kb. Probes on the array were 60-mers and covered both coding and non-coding regions of the human genome. A total of 1.0 µg genomic DNA from patients and controls was labeled with Cy3 and Cy5 dyes respectively using the CytoSure Genomic DNA labeling kit (Oxford Gene Technology). Probes were then purified using Microcon Centrifugation Filters, Ultracel YM-30 (Millipore, Billerica, MA, USA) and mixed thoroughly. This was followed by denaturation and pre-annealing with 50 µg human Cot-1 DNA (Invitrogen, California). Hybridization of the mixture to the array slide was executed at a constant rotation at 65°C for 40 hours. The slide was then washed with Agilent wash buffers 1 and 2, and scanned immediately using an Agilent Microarray scanner (Agilent Technologies, Santa Clara, CA, USA). Data were extracted from scanned images using Feature Extraction Software, version 10.7.3.1 (Agilent Technologies, USA). The raw data obtained thereafter were uploaded into the CytoSure Interpret software version 4.2.5 (Oxford Gene Technology), normalized and converted into.cgh files. Data normalization software was used to improve inconsistencies in dye incorporation. The data were segmented using a modified Circular Binary Segmentation (CBS) algorithm [27]. Genomic aberrations were identified by applying log2 intensity ratios of sample to reference (Cy3/Cy5: log2-ratios above 0.3 for duplications and below −0.6 for deletions). Chromosomal aberrations were reported in accordance with the human genome sequence assembly Build 37, hg 19 (http://www.ncbi.nlm.nih.gov). The microarray data have been deposited in the Gene Expression Omnibus (GEO) database (accession number 55645).

### CNV Calling and Functional Enrichment Analysis

CNVs were called for the segments with at least 5 consecutive probes. Rare CNVs were defined as those which overlapped by <50% with reported CNVs from the Database of Genomic Variants (DGV; http://dgv.tcag.ca/dgv/app/home). CNVs were deemed to be novel if they did not appear in the DGV database. Gene content within the identified CNVs was retrieved from the *Homo sapiens* (GRCh27) assembly using the Biomart-Ensembl (http://www.ensembl.org). By default, the lists contained both gene and non-gene entities; the latter were removed through a process of cross-checking and verification of gene symbols using the HUGO Gene Nomenclature Committee (HGNC) database (http://www.genenames.org/). To investigate the functional impact of rare and/or novel CNVs, the Database for Annotation, Visualization and Integrated Discovery (DAVID; http://david.abcc.ncifcrf.gov/) was utilised to assess the Gene Ontology (GO; http://www.geneontology.org/) and Kyoto Encyclopedia of Genes and Genomes (KEGG) pathway (http://www.genome.jp/kegg/) annotations between the genes encompassing the rare and/or novel CNVs and the genes associated with NAFLD. The list of genes associated with NAFLD was identified using the MalaCards database (http://www.malacards.org/) – an integrated searchable database of human disease states and their annotations, in association with the GeneCards relational database. Initially, a total of 200 genes associated with NAFLD were identified. Since the gene-disease association was based on a text mining algorithm, a manual verification of the biological processes associated with each of the 200 genes was performed. Only genes that had previously been described as being associated with NAFLD by either expression studies, genotyping or protein array work were selected, thereby lowering the number of genes implicated in NAFLD to 70 (see Table S1 for the complete list of genes).

### Quantitative PCR Validation of CNV Calls

A duplex TaqMan real-time quantitative polymerase chain reaction (qPCR) was performed to validate the CNV regions using a Step One Plus (Applied Biosystems) on three of the samples from two selected regions (11q11: Assay Hs02799097_cn, and 13q12.11: Assay Hs03857719_cn). Each reaction (20 µL) contained 10 µL master mix, 1 µL TaqMan Copy Number Assay, 1 µL TaqMan Copy Number Reference Assay, 4 µL nuclease free water, and 4 µL 5 ng/µL genomic DNA, and was run in quadruplicate. The PCR cycling conditions consisted of 1 PCR cycle at 95°C for 10 min, followed by 40 cycles at 95°C for 15 sec and 60°C for 1 min.

## Results

In the aCGH method adopted, DNA samples pooled from multiple subjects (patients and controls) were cross-compared so as to remove/normalise any common copy number changes in the normal control sample. Since the principle of aCGH is to compare the DNA copy number from patient samples against those of normal controls, CNV calls were designed to be patient-specific.

### Subjects and Identification of CNVs in the NASH Genome

**Figure 1:**
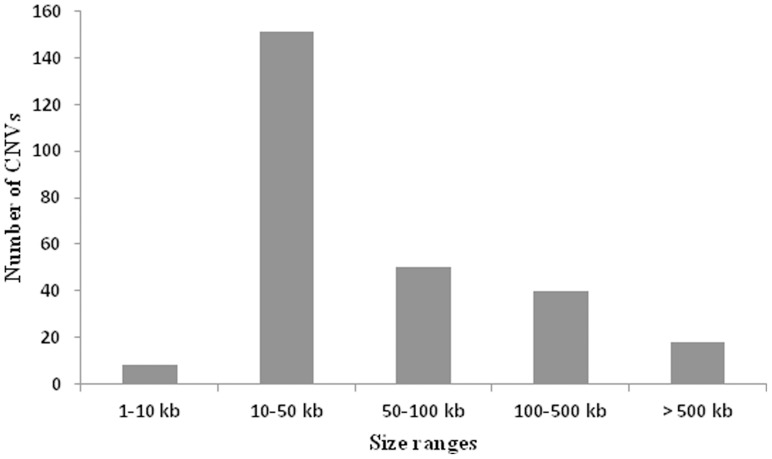
Size range distribution of the CNVs. The CNVs ranged in size from 5.77

All DNA samples passed quality control (QC) after a rigorous sample preparation process and a QC check during sample processing. Sets of 39 NASH samples and 39 matched controls were run in parallel on an array CGH platform that allowed the ratio of DNA copy number between a test (patient) and a reference (control) to be simultaneously assessed. From a total of 39 samples, 51.3% (*n* = 20) were females, 48.7% (n = 19) were males; the mean age of the 39 subjects was 50.4 years. The histopathological data are presented in Table 1. Seven percent of CNV calls were attributable to the sex chromosomes (with a frequency of at least 10%), but we opted to exclude these chromosomes from further analysis owing to the evolutionary biases due to small imbalances of the sex chromosomes [28]. Analysis of copy number variants, on the basis of log ratio and probe incidence filtering, yielded a total of 267 autosomal CNVs (the ratio of the fluorescence intensities between the patients and controls is a measure of the relative DNA copy number), amounting to an average of 6.84 autosomal CNVs per individual. The 267 CNVs detected spanned between 5.77 kb and 8.15 Mb in size, with a mean size of 194.94 kb and a median size of 38.33 kb, covering a total of 52.05 Mb or 1.63% of the genome (Fig. 1). Most chromosomal arms harboured both copy number gains and losses, but copy number gains were more commonly observed than losses (estimated ratio of 1.7∶1). However, only 55 CNVs (20.6%) out of the 267 CNVs detected had a frequency of >10%.

**Table 1:** Histopathological data in patients with NAFLD.

	NASH (n = 39)	Simple steatosis (n = 10)
Steatosis grade		
1	11	9
2	20	1
3	8	0
Inflammatory activity		
0	1	3
1	19	7
2	19	0
3	0	0
Ballooning		
0	0	10
1	21	0
2	18	0
Fibrosis stage		
0	0	10
1	12	0
2	19	0
3	6	0
4	2	0

NASH, non-alcoholic steatohepatitis.

Molecular genomic profiling identified 14q11.2 as the most frequently amplified region, which occurred in 53.8% of the NASH samples and contained a clutch of olfactory receptor (*OR*) family genes (Table 2; see Table S2 for the full list of *OR* genes). The most frequently deleted genomic region in the NASH samples, 12p13.2, is enriched in taste receptor (*TASR*) family genes (see Table S2 for the full list of *TASR* genes), and exhibited similar frequencies of losses and gains (38.5%) suggesting a generally unstable region. Several other frequently deleted regions were also observed including one at 16q12.2 harbouring the carboxylesterase 1 (*CES1*) gene and one at 14q24.3 spanning the acyl-CoA thioesterase 1 (*ACOT1*) gene; importantly, both genes are known to promote hepatic steatosis via the action of regulation of hepatic lipid metabolism [29]–[30]. There were nine CNVs present in at least 33% of the samples whilst only one was present in at least half of the samples. Six CNVs occurred with a frequency of at least 10% in samples which contained copy number duplications in the chromosomal regions 16p12.2 (27.5%), 12q24.33 (12.8%), 22q13.2 (12.8%), 12q13.2 (10.3%), 2q37.1 (10.3%) and 21p11.2-p11.1 (10.3%), implying that these regions could be involved in the development of NASH. Overall, nearly 50% of genomic regions were reported only as copy number gains; however, only 6 of these regions were present at a frequency of more than 10%. By contrast, about 18% of the genomic regions presented only as losses; however, none had a frequency greater than 10%.

**Table 2:** Top regions of copy number gains and losses in NASH.

Cytoband	Samplefrequency (%)	DGVcoverage (%)	Start	End	Size (kb)	Number of geneswithin demarcatedregion	Candidategene (s)
					Amplification		
14:q11.2	53.8	100	19,728,641	20,420,849	692.21	15	*OR*
5:p15.33	41	100	723,194	820,424	97.23	3	–
11:p15.4	35.9	100	5,893,184	5,935,144	41.96	3	*OR*
12:p13.31	33.3	100	9,637,323	9,718,846	81.52	0	–
					Deletion		
11:q11	35.9	100	55,368,154	55,450,788	82.63	6	*OR*
14:q24.3	33.3	100	74,001,651	74,022,324	20.67	3	*ACOT1*
16:q12.2	33.3	100	55,832,511	55,853,358	20.85	1	*CES1*
4:q13.2	33.3	100	69,392,545	69,483,277	90.73	1	–
					Amplification &Deletion		
12:p13.2	38.5	100	11,219,788	11,249,210	29.42	4	*TASR*

Start = first base-pair location in the copy number region, End = last base-pair location in the copy number region.

### Integrative Analysis of CNVs and Functional Enrichment to Identify Candidate Genes for Involvement in NASH

To identify unique CNVs in NASH patients that could be involved in the pathogenesis of this condition, we performed a cross-comparison with known CNVs from the DGV database. Conservative assessment of the overlap between reported CNVs from the DGV database with the CNVs identified in this study revealed four rare and/or novel CNVs (DGV coverage <50%) that were present in at least 10% of the NASH samples (Table 3). Two of these CNVs were classed as rare (DGV coverage <50%: 12q24.33 and 13q12.11), whereas the other two were novel (DGV coverage 0%: 21p11.1–11.2 and 12q13.2). A Chi-square test confirmed the significance of the association of these CNVs with NASH (*P*<0.05) as compared to simple steatosis. To further assess the likelihood of the involvement of these CNVs in NASH, the genes located within these regions were identified and their involvement in those biological processes shared with known NAFLD genes assessed. First, we profiled the genes within the chromosomal regions that are bounded by the four rare and/or novel CNVs, where genes such as exportin 4 (*XPO4*) and phosphodiesterase 1B (*PDE1B*) are located. A list of genes known to be associated with NAFLD was then obtained (see Table S1). Subsequently, we performed GO enrichment and KEGG pathway analysis using the DAVID gene annotation tool for the two sets of genes (genes within the four unique regions and known genes associated with NAFLD). We observed a number of shared biological processes (Table 4) between the two sets of genes including those that could be linked to NAFLD progression such as glucose metabolism, cell surface receptor-linked signal transduction and cell death [3].

**Table 3:** Rare and novel CNVs in NASH.

Cytoband	Samplefrequency (%)	DGVcoverage (%)	Start	End	Size (kb)	Number of geneswithin demarcatedregion	CandidateGene (s)
					Amplification		
*Rare*							
12q24.33	12.8	<50	131,432,076	131,460,728	28.65	1	–
13q12.11	10.3	<50	21,475,933	21,494,311	18.38	1	*XPO4*
*Novel*							
21p11.1–11.2	10.3	0	10,347,806	10,944,060	596.25	5	–
12q13.2	10.3	0	54,962,801	54,983,141	20.34	2	*PDE1B*

Start = first base-pair location in the copy number region, End = last base-pair location in the copy number region.

**Table 4:** Enriched GO terms associated with NASH.

Category	Term	Count	Involved genes/total genes (%)	*P*-Value*
GO_CC	GO:0005576∼extracellular region	34	44.16	1.2E-10
GO_BP	GO:0006006∼glucose metabolic process	6	7.79	2.0E-04
GO_BP	GO:0019318∼hexose metabolic process	6	7.79	6.8E-04
GO_BP	GO:0005996∼monosaccharidemetabolic process	6	7.79	1.4E-03
GO_BP	GO:0015980∼energy derivationby oxidation of organic compounds	4	5.19	8.3E-03
GO_BP	GO:0007186∼G-protein coupledreceptor protein signaling pathway	12	15.58	2.0E-02
GO_BP	GO:0007166∼cell surface receptorlinked signal transduction	16	20.78	4.6E-02
GO_BP	GO:0006091∼generation ofprecursor metabolites and energy	5	6.49	3.0E-02
GO_BP	GO:0008219∼cell death	8	10.39	4.5E-02

GO_BP, Gene Ontology biological process; GO_CC, Gene Ontology cellular component.

*Modified Fisher’s Exact test, *P*-Value ≤0.05.

### Identification of CNVs in the Simple Steatosis Genome

Given the greater number of NASH samples (∼80%) and the progressive nature of NASH (about one third of NASH patients tend to develop cirrhosis over a 5–10 year period; by contrast, simple steatosis patients tend to be clinically stable over time) [31] in the disease spectrum, the main focus of this study was placed on NASH. However, we were also interested in understanding the progression of simple steatosis to NASH. Unfortunately, we were only able to obtain DNA samples from 10 simple steatosis patients and 10 fatty-liver free controls. Seven of the samples were male and the mean age (all samples) was 47.9. The histopathological data are shown in Table 1. A total of 56 CNVs (simple steatosis patient-specific) were identified, including three (5.4%) which were located on one of the sex chromosomes. All CNVs were present with a frequency of at least 10%. Fifty-three autosomal CNVs were selected for further analysis. Of these, 11 were unique to simple steatosis whereas 42 were found to be shared with NASH. The former 11 CNVs could conceivably play a role in the development of hepatic steatosis, whereas the latter 42 CNVs could be involved in progression to steatohepatitis. Intriguingly, the four rare and/or novel CNVs identified earlier in NASH patients were not found in simple steatosis patients, and remain unique to NASH.

The top scoring regions in terms of copy number gains and losses in simple steatosis are listed in Table 5. The most commonly amplified region, 12p13.31 (50%), was also among the most highly amplified regions observed in NASH patients. A CNV at the 10q11.22 locus that occurred in 40% of the simple steatosis samples contains the neuropeptide Y receptor 4 (*NPYR4*) gene, which is known to be important in obesity through the regulation of appetite and energy metabolism [32]. Three CNVs (located at 4q13.2, 15q11.2 and 11q11) shared the most deleted region at a frequency of 40%, in which two of the CNVs (4q13.2 and 11q11) were also among the most highly deleted regions observed in NASH. These CNVs were enriched for *OR* genes (11q11) and immunoglobulin heavy chain (*IGH*) (15q11.2) family genes (Table 4; see Table S2 for the full list of *OR* and *IGH* genes). However, all CNVs identified in the simple steatosis patients were common (DGV coverage 100%).

**Table 5:** Top regions of copy number gains and losses in simple steatosis.

Cytoband	Samplefrequency (%)	DGVcoverage (%)	Start	End	Size (kb)	Number of geneswithin demarcatedregion	Candidategene (s)
					Amplification		
12:p13.31	50	100	9,637,323	9,718,846	81.52	0	–
10:q11.22	40	100	46,971,647	47,394,442	422.80	14	*NPY4R*
16:p13.11	40	100	15,048,676	15,120,666	71.99	2	–
8:p11.23	40	100	39,234,992	39,386,158	151.17	0	–
					Deletion		
11:q11	35.9	100	55,368,154	55,450,788	82.63	6	*OR*
15:q11.2	33.3	100	20,481,702	22,578,630	2096.93	32	*OR, IGH*
					Amplification &Deletion		
4:q13.2	40	100	69,392,545	69,483,277	90.73	1	–

Start = first base-pair location in the copy number region, End = last base-pair location in the copy number region.

### qPCR Validation

**Figure 2:**
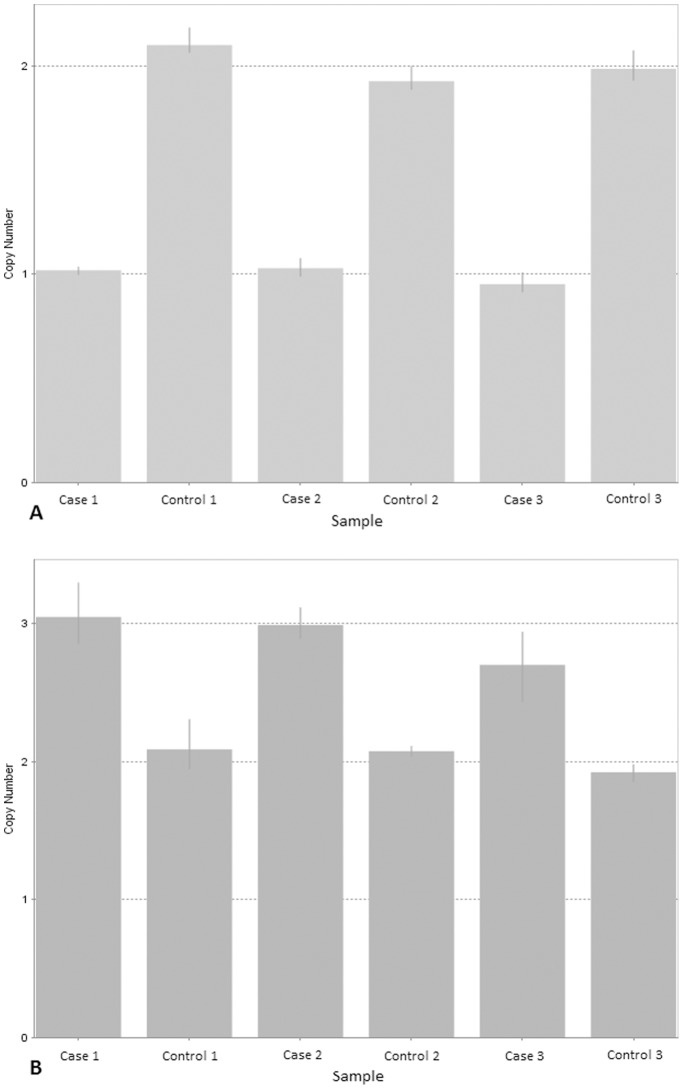
qPCR validation performed on selected genomic regions. (**A**) Results for the region 11q11. (**B**) Results for the region 13q12.11. A copy number of around 2 was deemed to be indicative of wild-type status (i.e. no CNV), a copy number of 1 was indicative of one copy lost, whereas a copy number of 3 or above was held to indicate copy number gain(s). The error bars represent the standard error among four replicates.

We validated three samples (each is patient-control matched pair) for each CNV region identified. We selected two CNV regions that represented different statuses of copy number change (the CNV at 13q12.11 was a copy number gain and was rare, 11q11 was a copy number loss). All CNVs were confirmed through qPCR validation. Amplifications and deletions of the genomic regions were defined on the basis of differences between patient’s copy number and the wild-type copy number (i.e. a copy number around 2). Fig. 2 illustrates the qPCR results of the validated CNVs.

## Discussion

Studies on CNVs are becoming increasingly important in studies of inherited disease, with growing evidence attesting to the substantial impact that they can have on human phenotypic variability and genetic susceptibility. Here, we present a pilot analysis of CNVs in a series of NAFLD patients. We identified four CNVs that are either rare or novel to NASH patients in our study that could potentially contribute to clinical outcome.

In patients with NASH, the most frequently amplified region was 14q11.2, which is enriched in *OR* family genes, while an abundance of *TASR* family genes were found at 12p13.2, the most frequently deleted region. Although the *OR* and *TASR* families play roles in the olfactory and gustatory systems respectively, a search of the database of Expressed Sequence Tags, NCBI dbEST, revealed *OR* and *TASR* gene expression in many tissues and organs, including the liver. Impairment of olfactory and gustatory function has been reported in chronic liver disease including cirrhosis; chemosensory function however improved after liver transplantation [33]. In the early 2000s, a comprehensive database of the human olfactory subgenome was completed using a highly automated data mining system [34]. Glusman et al. (2001) reported the presence of 906 potential coding regions for *OR* genes that cover almost all human chromosomes with the exception of chromosomes 20 and Y, in which 2/3 of the regions have not been reported. Subsequently, new databases termed respectively the Olfactory Receptor Microarray Database (ORMD) which includes microarray gene expression data from the *ORs* [35], and the Database of Chemosensory Receptor Gene Families (CRDB), were developed [36]. The size of these databases highlights the importance of *OR* and *TASR* gene families not only in the olfactory and gustatory systems, but also in tissues and organs throughout the body.

A deletion CNV was noted at the 16q12.2 locus; it includes the *CES1* gene, which is primarily important in the metabolism of fatty acids and cholesterol [37]. Expression of *CES1* has been found to be higher in human NAFLD hepatic tissue as compared to non-NAFLD [38]. A role for *CES1* in lipolysis was evidenced by a positive correlation between *CES1* expression and triglyceride lipase activity as well as with adiposity [30]. On the other hand, *CES1* knockout mice are characterized by a gain in weight, hepatic steatosis and hyperinsulinemia, thereby supporting a role for *CES1* in the regulation of fatty acids [37]. Interestingly, the 16q12.2 locus is known to harbour genetic variants (SNPs) associated with BMI [39]. Although *CES1* has been implicated in hepatic steatosis [37], a recent study has shown that CES1 may have potential as a biomarker to distinguish hepatocellular carcinoma (HCC) from cirrhosis [40]. Also notable among the highly deleted regions in NASH patients was a copy number loss at the 14q24.3 locus, where the acyl-CoA thioesterase 1 (*ACOT1*) gene resides. Acyl-CoA thioesterase 1 promotes the cellular balance between free fatty acids and acyl-CoAs to maintain cellular processes including lipid metabolism [29]. Compared to other *ACOT* subfamily genes, *ACOT1* is unique in that it is highly expressed only in association with a high fat diet but not in association with a normal diet [41].

Although determining the CNV frequencies and their gene content are important, most of the CNVs detected here are considered to be common (DGV coverage 100%) and hence may have little or no impact on the pathogenesis of NASH. The definition of ‘common’ here is however debateable given that reported CNVs from the DGV are (i) from non-NAFLD studies and (ii) unlikely to be from the Malay population. Caution should therefore be exercised when offering functional interpretation of these CNVs until more comprehensive studies on larger numbers of patients are conducted. To achieve our main goal in this pilot study, which was to identify candidate CNV loci that could play a role in the etiology of NASH, we filtered out common CNVs and identified four CNVs (DGV coverage <50%) that have the potential to be involved in the pathogenesis of NASH; two of these are rare (12q24.33 and 13q12.11) whilst two are novel (12q13.2 and 21p11.1–11.2). We were able to establish the potential significance of these loci by performing a Chi-square test against other loci and validating the findings by qPCR; in this way, we were able to confirm that, despite the relatively small sample size, our analysis has the potential to yield biologically meaningful and reproducible results. We postulate that these CNVs could provide new insights into the biology of NASH. Of particular note was an aberration at the 13q12.11 locus that could serve as a potential copy number biomarker for NASH. This region contains the tumor suppressor gene exportin 4 (*XPO4*), the inactivation of which promotes HCC in mice [42]. On the other hand, increased expression of *XPO4* in human HCC is associated with better prognosis and a better survival rate [43]–[44]. The phosphodiesterase 1B, calmodulin-dependent (*PDE1B*) gene spanning the 12q13.2 region is important in many signal transduction pathways, and has been found to be downregulated in cirrhotic liver [45]. The 12q13.2 locus was identified as a clear-cut amplification (no deletion event), thereby supporting its candidacy as a potential risk marker CNV associated with the disease. However, there are a limited number of published reports on *XPO4* and *PDE1B* and their putative role in liver disease. Thus, additional comprehensive studies focussed on these two genes will be necessary to confirm or refute this finding.

To assess the plausibility of our results, it was important to verify the functional role of these CNVs (rare/novel) and their potential impact on NASH. In order to explore the possible association between these CNVs and NASH, we extended our analysis to GO functional enrichment and KEGG pathway analysis for genes residing at these CNV loci and known NAFLD genes. The results yielded several shared biological processes between the two sets of genes. Of primary importance are glucose metabolism and cell surface receptor-linked signal transduction and cell death, all of which have been shown to be important in the pathogenesis of NASH [4]. However, no related KEGG pathway was observed.

As for simple steatosis, the most frequently amplified region (12p13.31) also happened to be among the most highly amplified regions in NASH. The 10q11.22 region, which harbours a CNV that occurred in 40% of the simple steatosis samples, contains the *NPYR4* gene. This gene is involved in the regulation of appetite and energy metabolism [32]. The pancreatic peptide, a high affinity ligand for the neuropeptide Y receptor 4 (Y4), has been suggested to have anti-obesity potential [46]–[47]. Long term antagonism of Y4 causes significant reduction in body weight and adiposity via effects on metabolic rate and energy distribution [48].

We readily acknowledge the small number of simple steatosis samples in the present study. This limitation was due to the lack of availability of simple steatosis patients from our previous study that comprised three major ethnic groups [49]–[50]. These patients were recruited from the UMMC, a tertiary referral center, which could explain the greater number of NASH patients as compared to those with simple steatosis. In order to minimise ethnicity as a potential confounding factor, we selected samples taken from only one specific ethnic group, namely the Malays, for both the NASH and the simple steatosis group. Under these conditions, the number of simple steatosis patients that we were able to obtain was only 10. Despite the limited numbers of patients available, several of our findings were statistically significant. Importantly, chromosome 11q11 which was one of the most frequently deleted CNVs in our study, was also frequently deleted from 10 hepatic steatosis patients from the study by Royo et al. [51]. It should be noted that the Royo et al. study did not include any NASH patients. This notwithstanding, our pilot study was designed to provide an initial screen of the structural genomic aberrations present in NAFLD samples. Simple steatosis patients mostly presented with either a copy number gain or a loss event at one locus, unlike the NASH group which tended to exhibit both events. In addition, a greater number of CNVs were identified in the NASH group as compared to the simple steatosis group. This could be explained by the complex pathogenesis of NAFLD especially at the NASH stage, involving not only the ‘first hit’ mechanism but also the ‘second hit’ [4]. In this study, we were mainly concerned with identifying CNVs that were common to both simple steatosis and NASH, particularly when the CNV frequency was higher in NASH than in simple steatosis (*n* = 2), as they could indicate involvement in the progression of the disease. Surprisingly, histological data from the samples harbouring these CNVs (12p13.2 and 11p15.4) showed a higher frequency (53.3% and 71.4% respectively) of fibrosis score ≥2, thereby supporting the disease progression model.

The ethical issue that precluded the use of liver biopsy for the classification of controls (non-NAFLD) required us to adopt a stringent definiton of controls in order to rule out fatty liver in the control subjects; biochemical tests, ultrasonography and MRI evaluations were therefore used to minimise misclassification of our controls. To the best of our knowledge, this is the first study to investigate a genome-wide profile of copy number variation in the NAFLD spectrum; hence, determination of the CNV total number, frequency, genomic location and gene content, is challenging. The use of aCGH technology allows CNV discovery at high resolution and hence allows confidence in CNV detection. The use of 60mer probes provides high sensitivity and specificity to accurately detect both known and *de novo* CNVs as compared to shorter oligonucleotide probes [52]. The source of genes known to be related to NAFLD was Malacard, which is known to use a text-mining approach [53]. Hence, a manual verification of the gene functions was performed that included only genes that have been shown to be associated in either expression studies, genotyping or protein array work. However, we cannot rule out the possibility that other genes could be of importance in NAFLD, as more comprehensive studies are still ongoing. Indeed, it was also difficult for us to assess the significance of such CNVs given that multiple genes often reside within the CNV intervals. We attempted to overcome this limitation by performing a functional enrichment analysis that covered all the genes residing within the CNV regions.

Taken together, the results of our whole genome copy number analysis have documented four rare and/or novel CNV loci that are unique to NASH, and to the best of our knowledge, have not previously been reported. This study nevertheless falls into the hypothesis generating category rather than the hypothesis testing category; hence, our results remain to be substantiated by additional studies on larger patient groups. Moreover, additional functional studies on the genes residing within these loci will be needed to fully characterize the function of the genes and their relationship, if any, to NASH.

## Supporting Information

Table S1 **List of genes known to be associated with NAFLD.**
(XLSX)

Table S2 **CNV regions with candidate genes.**
(XLSX)

## References

[1] Fabbrini E, Sullivan S, Klein S (2010) Obesity and nonalcoholic fatty liver disease: biochemical, metabolic, and clinical implications. Hepatology 51: 679–689. 20041406 10.1002/hep.23280 PMC3575093

[2] Malaguarnera M, Di Rosa M, Nicoletti F, Malaguarnera L (2009) Molecular mechanisms involved in NAFLD progression. J Mol Med (Berl) 87: 679–695. 19352614 10.1007/s00109-009-0464-1

[3] Jou J, Choi SS, Diehl AM (2008) Mechanisms of disease progression in nonalcoholic fatty liver disease. Semin Liver Dis 28: 370–379. 18956293 10.1055/s-0028-1091981

[4] Farrell GC, van Rooyen D, Gan L, Chitturi S (2012) NASH is an inflammatory disorder: pathogenic, prognostic and therapeutic implications. Gut Liver 6: 149–171. 22570745 10.5009/gnl.2012.6.2.149 PMC3343154

[5] Chalasani N, Younossi Z, Lavine JE, Diehl AM, Brunt EM, et al (2012) The diagnosis and management of non-alcoholic fatty liver disease: practice guideline by the American Gastroenterological Association, American Association for the Study of Liver Diseases, and American College of Gastroenterology. Gastroenterology 142: 1592–1609. 22656328 10.1053/j.gastro.2012.04.001

[6] Ekstedt M, Franzen LE, Mathiesen UL, Thorelius L, Holmqvist M, et al (2006) Long-term follow-up of patients with NAFLD and elevated liver enzymes. Hepatology 44: 865–873. 17006923 10.1002/hep.21327

[7] Hernaez R (2012) Genetic factors associated with the presence and progression of nonalcoholic fatty liver disease: a narrative review. Gastroenterol Hepatol 35: 32–41. 22093607 10.1016/j.gastrohep.2011.08.002

[8] Farrell GC, Wong VW, Chitturi S (2013) NAFLD in Asia–as common and important as in the West. Nat Rev Gastroenterol Hepatol 10: 307–318. 23458891 10.1038/nrgastro.2013.34

[9] Vernon G, Baranova A, Younossi ZM (2011) Systematic review: the epidemiology and natural history of non-alcoholic fatty liver disease and non-alcoholic steatohepatitis in adults. Aliment Pharmacol Ther 34: 274–285. 21623852 10.1111/j.1365-2036.2011.04724.x

[10] Schwimmer JB, Celedon MA, Lavine JE, Salem R, Campbell N, et al (2009) Heritability of nonalcoholic fatty liver disease. Gastroenterology 136: 1585–1592. 19208353 10.1053/j.gastro.2009.01.050 PMC3397140

[11] Romeo S, Kozlitina J, Xing C, Pertsemlidis A, Cox D, et al (2008) Genetic variation in PNPLA3 confers susceptibility to nonalcoholic fatty liver disease. Nat Genet 40: 1461–1465. 18820647 10.1038/ng.257 PMC2597056

[12] Speliotes EK, Yerges-Armstrong LM, Wu J, Hernaez R, Kim LJ, et al (2011) Genome-wide association analysis identifies variants associated with nonalcoholic fatty liver disease that have distinct effects on metabolic traits. PLoS Genet 7: e1001324. 21423719 10.1371/journal.pgen.1001324 PMC3053321

[13] Iafrate AJ, Feuk L, Rivera MN, Listewnik ML, Donahoe PK, et al (2004) Detection of large-scale variation in the human genome. Nat Genet 36: 949–951. 15286789 10.1038/ng1416

[14] Sebat J, Lakshmi B, Troge J, Alexander J, Young J, et al (2004) Large-scale copy number polymorphism in the human genome. Science 305: 525–528. 15273396 10.1126/science.1098918

[15] Redon R, Ishikawa S, Fitch KR, Feuk L, Perry GH, et al (2006) Global variation in copy number in the human genome. Nature 444: 444–454. 17122850 10.1038/nature05329 PMC2669898

[16] Pinto D, Marshall C, Feuk L, Scherer SW (2007) Copy-number variation in control population cohorts. Hum Mol Genet 16 Spec No. 2: R168–173. 10.1093/hmg/ddm241 17911159

[17] Stone JL, O'Donovan MC, Gurling H, Kirov GK, Blackwood DH, et al (2008) Rare chromosomal deletions and duplications increase risk of schizophrenia. Nature 455: 237–241. 18668038 10.1038/nature07239 PMC3912847

[18] Pinto D, Pagnamenta AT, Klei L, Anney R, Merico D, et al (2010) Functional impact of global rare copy number variation in autism spectrum disorders. Nature 466: 368–372. 20531469 10.1038/nature09146 PMC3021798

[19] Lionel AC, Crosbie J, Barbosa N, Goodale T, Thiruvahindrapuram B, et al (2011) Rare copy number variation discovery and cross-disorder comparisons identify risk genes for ADHD. Sci Transl Med 3: 95ra75. 10.1126/scitranslmed.3002464 21832240

[20] Clarke AJ, Cooper DN (2010) GWAS: heritability missing in action? Eur J Hum Genet 18: 859–861. 20234388 10.1038/ejhg.2010.35 PMC2987389

[21] Bae JS, Cheong HS, Kim JH, Park BL, Park TJ, et al (2011) The genetic effect of copy number variations on the risk of type 2 diabetes in a Korean population. PLoS One 6: e19091. 21526130 10.1371/journal.pone.0019091 PMC3081314

[22] Wheeler E, Huang N, Bochukova EG, Keogh JM, Lindsay S, et al (2013) Genome-wide SNP and CNV analysis identifies common and low-frequency variants associated with severe early-onset obesity. Nat Genet 45: 513–517. 23563609 10.1038/ng.2607 PMC4106235

[23] Brunt EM, Kleiner DE, Wilson LA, Belt P, Neuschwander-Tetri BA (2011) Nonalcoholic fatty liver disease (NAFLD) activity score and the histopathologic diagnosis in NAFLD: distinct clinicopathologic meanings. Hepatology 53: 810–820. 21319198 10.1002/hep.24127 PMC3079483

[24] Kleiner DE, Brunt EM, Van Natta M, Behling C, Contos MJ, et al (2005) Design and validation of a histological scoring system for nonalcoholic fatty liver disease. Hepatology 41: 1313–1321. 15915461 10.1002/hep.20701

[25] Ruhl CE, Everhart JE (2005) Joint effects of body weight and alcohol on elevated serum alanine aminotransferase in the United States population. Clin Gastroenterol Hepatol 3: 1260–1268. 16361053 10.1016/s1542-3565(05)00743-3

[26] Sanyal AJ (2002) AGA technical review on nonalcoholic fatty liver disease. Gastroenterology 123: 1705–1725. 12404245 10.1053/gast.2002.36572

[27] Venkatraman ES, Olshen AB (2007) A faster circular binary segmentation algorithm for the analysis of array CGH data. Bioinformatics 23: 657–663. 17234643 10.1093/bioinformatics/btl646

[28] Nguyen DQ, Webber C, Hehir-Kwa J, Pfundt R, Veltman J, et al (2008) Reduced purifying selection prevails over positive selection in human copy number variant evolution. Genome Res 18: 1711–1723. 18687881 10.1101/gr.077289.108 PMC2577867

[29] Chang ML, Yeh CT, Chen JC, Huang CC, Lin SM, et al (2008) Altered expression patterns of lipid metabolism genes in an animal model of HCV core-related, nonobese, modest hepatic steatosis. BMC Genomics 9: 109. 18307821 10.1186/1471-2164-9-109 PMC2287171

[30] Nagashima S, Yagyu H, Takahashi N, Kurashina T, Takahashi M, et al (2011) Depot-specific expression of lipolytic genes in human adipose tissues - association among CES1 expression, triglyceride lipase activity and adiposity. J Atheroscler Thromb 18: 190–199. 21081832 10.5551/jat.6478

[31] Caldwell S, Argo C (2010) The natural history of non-alcoholic fatty liver disease. Dig Dis 28: 162–168. 20460906 10.1159/000282081

[32] Herzog H (2003) Neuropeptide Y and energy homeostasis: insights from Y receptor knockout models. Eur J Pharmacol 480: 21–29. 14623347 10.1016/j.ejphar.2003.08.089

[33] Bloomfeld RS, Graham BG, Schiffman SS, Killenberg PG (1999) Alterations of chemosensory function in end-stage liver disease. Physiol Behav 66: 203–207. 10336145 10.1016/s0031-9384(98)00266-2

[34] Glusman G, Yanai I, Rubin I, Lancet D (2001) The complete human olfactory subgenome. Genome Res 11: 685–702. 11337468 10.1101/gr.171001

[35] Liu N, Crasto CJ, Ma M (2007) Integrated olfactory receptor and microarray gene expression databases. BMC Bioinformatics 8: 231. 17603910 10.1186/1471-2105-8-231 PMC1955752

[36] Dong D, Jin K, Wu X, Zhong Y (2012) CRDB: database of chemosensory receptor gene families in vertebrate. PLoS One 7: e31540. 22393364 10.1371/journal.pone.0031540 PMC3290609

[37] Quiroga AD, Li L, Trotzmuller M, Nelson R, Proctor SD, et al (2012) Deficiency of carboxylesterase 1/esterase-x results in obesity, hepatic steatosis, and hyperlipidemia. Hepatology 56: 2188–2198. 22806626 10.1002/hep.25961

[38] Ashla AA, Hoshikawa Y, Tsuchiya H, Hashiguchi K, Enjoji M, et al (2010) Genetic analysis of expression profile involved in retinoid metabolism in non-alcoholic fatty liver disease. Hepatol Res 40: 594–604. 20618457 10.1111/j.1872-034X.2010.00646.x

[39] Peters U, North KE, Sethupathy P, Buyske S, Haessler J, et al (2013) A systematic mapping approach of 16q12.2/FTO and BMI in more than 20,000 African Americans narrows in on the underlying functional variation: results from the Population Architecture using Genomics and Epidemiology (PAGE) Study. PLoS Genet 9: e1003171. 23341774 10.1371/journal.pgen.1003171 PMC3547789

[40] Na K, Jeong SK, Lee MJ, Cho SY, Kim SA, et al (2013) Human liver carboxylesterase 1 outperforms alpha-fetoprotein as biomarker to discriminate hepatocellular carcinoma from other liver diseases in Korean patients. Int J Cancer 133: 408–415. 23319432 10.1002/ijc.28020

[41] Almon RR, Dubois DC, Sukumaran S, Wang X, Xue B, et al (2012) Effects of high fat feeding on liver gene expression in diabetic goto-kakizaki rats. Gene Regul Syst Bio 6: 151–168. 10.4137/GRSB.S10371 PMC3516129 23236253

[42] Zender L, Xue W, Zuber J, Semighini CP, Krasnitz A, et al (2008) An oncogenomics-based in vivo RNAi screen identifies tumor suppressors in liver cancer. Cell 135: 852–864. 19012953 10.1016/j.cell.2008.09.061 PMC2990916

[43] Liang XT, Pan K, Chen MS, Li JJ, Wang H, et al (2011) Decreased expression of XPO4 is associated with poor prognosis in hepatocellular carcinoma. J Gastroenterol Hepatol 26: 544–549. 21332550 10.1111/j.1440-1746.2010.06434.x

[44] Zhang H, Wei S, Ning S, Jie Y, Ru Y, et al (2013) Evaluation of TGFbeta, XPO4, elF5A2 and ANGPTL4 as biomarkers in HCC. Exp Ther Med 5: 119–127. 23251252 10.3892/etm.2012.750 PMC3523953

[45] Lee S, Kim S (2007) Gene regulations in HBV-related liver cirrhosis closely correlate with disease severity. J Biochem Mol Biol 40: 814–824. 17927917 10.5483/bmbrep.2007.40.5.814

[46] Lin S, Shi YC, Yulyaningsih E, Aljanova A, Zhang L, et al (2009) Critical role of arcuate Y4 receptors and the melanocortin system in pancreatic polypeptide-induced reduction in food intake in mice. PLoS One 4: e8488. 20041129 10.1371/journal.pone.0008488 PMC2796177

[47] Liu YL, Semjonous NM, Murphy KG, Ghatei MA, Bloom SR (2008) The effects of pancreatic polypeptide on locomotor activity and food intake in mice. Int J Obes (Lond) 32: 1712–1715. 18779824 10.1038/ijo.2008.160

[48] Zhang L, Bijker MS, Herzog H (2011) The neuropeptide Y system: pathophysiological and therapeutic implications in obesity and cancer. Pharmacol Ther 131: 91–113. 21439311 10.1016/j.pharmthera.2011.03.011

[49] Zain SM, Mohamed R, Mahadeva S, Cheah PL, Rampal S, et al (2012) A multi-ethnic study of a PNPLA3 gene variant and its association with disease severity in non-alcoholic fatty liver disease. Hum Genet 131: 1145–1152. 22258181 10.1007/s00439-012-1141-y PMC3374090

[50] Zain SM, Mohamed Z, Mahadeva S, Rampal S, Basu RC, et al (2013) Susceptibility and gene interaction study of the angiotensin II type 1 receptor (AGTR1) gene polymorphisms with non-alcoholic fatty liver disease in a multi-ethnic population. PLoS One 8: e58538. 23484035 10.1371/journal.pone.0058538 PMC3590220

[51] Royo F, Zabala A, Paz N, Acquadro F, Echevarria JJ, et al (2013) Genome-wide analysis of DNA copy number changes in liver steatosis. Br J Med Med Res 3: 1773–1785.

[52] Curtis C, Lynch AG, Dunning MJ, Spiteri I, Marioni JC, et al (2009) The pitfalls of platform comparison: DNA copy number array technologies assessed. BMC Genomics 10: 588. 19995423 10.1186/1471-2164-10-588 PMC2797821

[53] Rappaport N, Nativ N, Stelzer G, Twik M, Guan-Golan Y, et al (2013) MalaCards: an integrated compendium for diseases and their annotation. Database (Oxford) 2013: bat018. 23584832 10.1093/database/bat018 PMC3625956

